# Supplementary material for: Mutations in PCYT2 disrupt etherlipid biosynthesis and cause a complex hereditary spastic paraplegia
Source: Brain. 2019 Oct 22;142(11):3382–97. doi: 10.1093/brain/awz291 (PMC6821184; doi:10.1093/brain/awz291)
Supplement: awz291_Supplementary_Data [file awz291_supplementary_data.zip › awz291-Suppl_data/Supplementary_Data4.zip!/Supplementary information 3 - fibroblast lipidomics.zip!/Fibroblast lipidomics/Patient1 vs Patient2/Statistical plots and data/OPLS-DA.pdf]

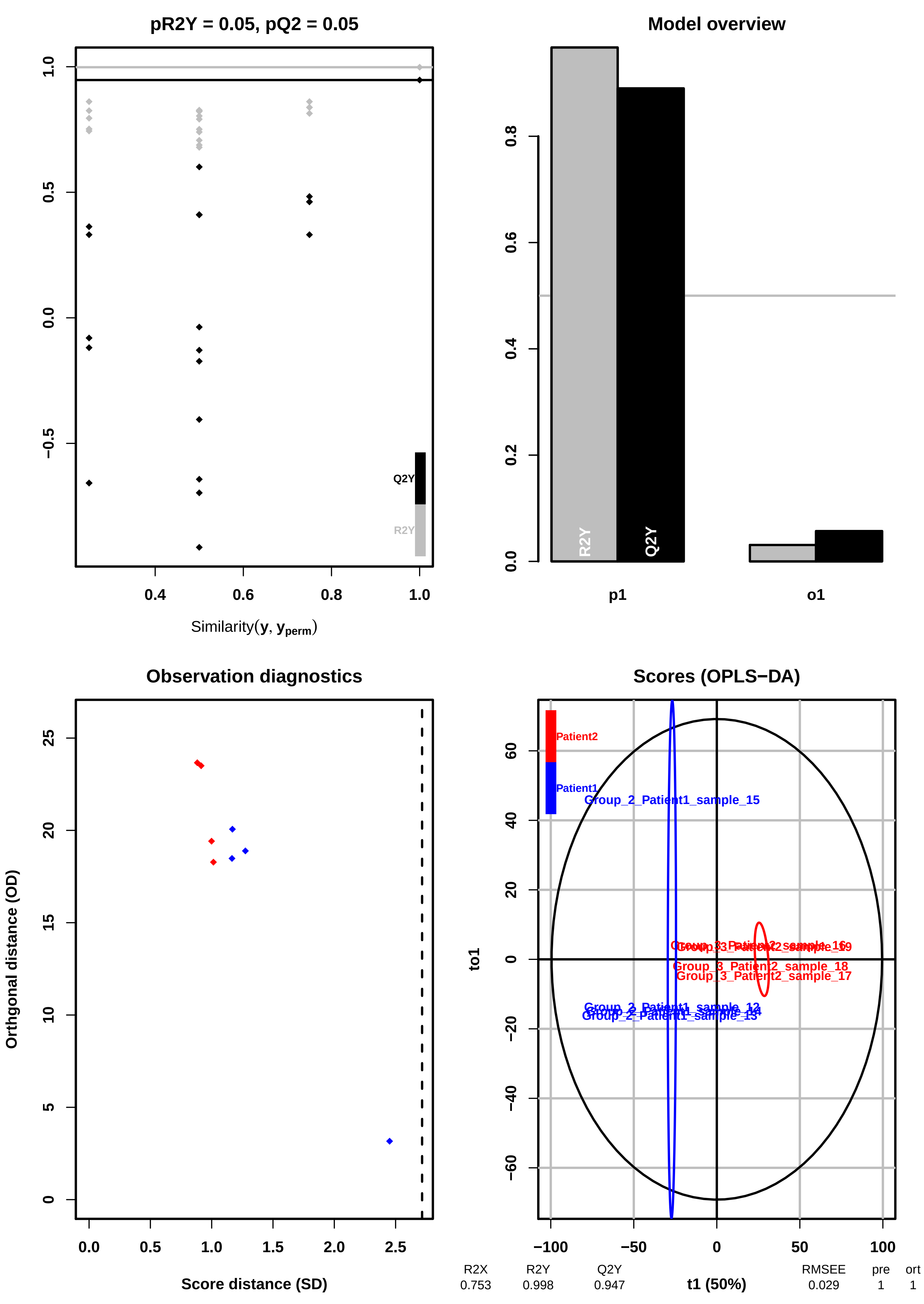
pR2Y = 0.05, pQ2 = 0.05
Similarity(y, yperm)
Q2Y
R2Y
Model overview
p1
o1
Observation diagnostics
Orthgonal distance (OD)
Score distance (SD)
Scores (OPLS−DA)
Patient2
Patient1
Group_2_Patient1_sample_15
Group_3_Patient2_sample_18
Group_3_Patient2_sample_17
Group_2_Patient1_sample_12
to1
t1 (50%)
R2X
0.753
R2Y
0.998
Q2Y
0.947
RMSEE
0.029
pre
1
ort
1
